# Generation of Persister Cells of *Pseudomonas aeruginosa* and *Staphylococcus aureus* by Chemical Treatment and Evaluation of Their Susceptibility to Membrane-Targeting Agents

**DOI:** 10.3389/fmicb.2017.01917

**Published:** 2017-10-04

**Authors:** Lucia Grassi, Mariagrazia Di Luca, Giuseppantonio Maisetta, Andrea C. Rinaldi, Semih Esin, Andrej Trampuz, Giovanna Batoni

**Affiliations:** ^1^Department of Translational Research and New Technologies in Medicine and Surgery, University of Pisa, Pisa, Italy; ^2^Charité – Universitätsmedizin Berlin, Corporate Member of Freie Universität Berlin, Humboldt-Universität zu Berlin, and Berlin Institute of Health, Berlin, Germany; ^3^Berlin-Brandenburger Centrum für Regenerative Therapien, Charité – Universitätsmedizin Berlin, Berlin, Germany; ^4^Department of Biomedical Sciences, University of Cagliari, Monserrato, Italy

**Keywords:** persisters, induction of persistence, *Staphylococcus aureus*, *Pseudomonas aeruginosa*, isothermal microcalorimetry, antimicrobial peptides

## Abstract

Persister cells (PCs) are a subset of dormant, phenotypic variants of regular bacteria, highly tolerant to antibiotics. Generation of PCs *in vivo* may account for the recalcitrance of most chronic infections to antimicrobial treatment and demands for the identification of new antimicrobial agents able to target such cells. The present study explored the possibility to obtain *in vitro* PCs of *Pseudomonas aeruginosa* and *Staphylococcus aureus* at high efficiency through chemical treatment, and to test their susceptibility to structurally different antimicrobial peptides (AMPs) and two clinically used peptide-based antibiotics, colistin and daptomycin. The main mechanism of action of these molecules (i.e., membrane-perturbing activity) renders them potential candidates to act against dormant cells. Exposure of stationary-phase cultures to optimized concentrations of the uncoupling agent cyanide *m*-chlorophenylhydrazone (CCCP) was able to generate at high efficiency PCs exhibiting an antibiotic-tolerant phenotype toward different classes of antibiotics. The metabolic profile of CCCP-treated bacteria was investigated by monitoring bacterial heat production through isothermal microcalorimetry and by evaluating oxidoreductase activity by flow cytometry. CCCP-pretreated bacteria of both bacterial species underwent a substantial decrease in heat production and oxidoreductase activity, as compared to the untreated controls. After CCCP removal, induced persisters showed a delay in heat production that correlated with a lag phase before resumption of normal growth. The metabolic reactivation of bacteria coincided with their reversion to an antibiotic-sensitive phenotype. Interestingly, PCs generated by CCCP treatment resulted highly sensitive to three different membrane-targeting AMPs at levels comparable to those of CCCP-untreated bacteria. Colistin was also highly active against PCs of *P. aeruginosa*, while daptomycin killed PCs of *S. aureus* only at concentrations 32 to 64-fold higher than those of the tested AMPs. In conclusion, CCCP treatment was demonstrated to be a suitable method to generate *in vitro* PCs of medically important bacterial species at high efficiency. Importantly, unlike conventional antibiotics, structurally different AMPs were able to eradicate PCs suggesting that such molecules might represent valid templates for the development of new antimicrobials active against persisters.

## Introduction

Within an isogenic bacterial population, PCs comprise a small subpopulation of non-growing, metabolically quiescent cells that exhibit high tolerance to antibiotics (Keren et al., 2004; Lewis, 2010). Unlike drug-resistant bacteria, PCs survive antibiotic treatments by reducing their metabolism and entering into a dormant state without undergoing genetic changes. Consequently, they do not proliferate in the presence of antibiotics, but they resume growth after the removal of the drug, giving rise to a population genetically identical to the original one and equally susceptible to antibiotics (Balaban et al., 2004; Kwan et al., 2013). Although PCs can arise from stochastic events in growing cultures (Maisonneuve et al., 2013), evidence suggests that their formation can also be induced as a response to several environmental factors, such as nutrients and oxygen deprivation, oxidative stress, DNA damage and antibiotics (Dörr et al., 2009; Wang et al., 2017). For instance, persister levels typically increase from exponential-phase cultures to stationary-phase cultures and biofilms, where PCs comprise as much as 1% of the total bacterial population (Lewis, 2008).

Persister cells represent a major challenge for the treatment of many types of infections. As they are present in significant number in biofilms, it is believed that they largely contribute to the recalcitrance to antibiotic treatment and chronicization of biofilm-associated infections (e.g., urinary tract infections, lung infections in cystic fibrosis, medical device-associated infections) (Lewis, 2008; Conlon, 2014). Indeed, while antibiotic treatment leads to the elimination of most of the biofilm-susceptible population, a small fraction of phenotypic persister variants survives. Once the antibiotic therapy is suspended, residual PCs resume growth and allow for the recolonization of the infection site, precluding the eradication of the infection (Lewis, 2001; Levin and Rozen, 2006). As the elimination of PCs seems crucial to improve the management of chronic biofilm-associated infections, the development of novel antimicrobial agents able to target such cells is emerging as a research priority (Conlon et al., 2013; Zhang, 2014).

The low frequency of PCs and their transient nature make it difficult to isolate the persister population from bacterial cultures, thereby representing a substantial limitation for the study of persistence and the identification of anti-persister molecules (Cañas-Duarte et al., 2014). Recent studies have demonstrated the possibility to obtain bacterial populations with high levels of PCs through chemical treatments. In particular, Kwan et al. (2013) have found that the uncoupling agent CCCP is able to efficiently induce persistence in cultures of *Escherichia coli*. CCCP has been reported to decrease ATP production by bacterial ATPases through the dissipation of the proton motive force, thereby leading to a drop in cell metabolic activity (Strahl and Hamoen, 2010). Although multiple mechanisms underlie persister formation, reduction in ATP levels has been lately proposed as a universal cause of multidrug tolerance in bacteria since the activity of most bactericidal antibiotics is directed toward targets expressed by metabolically active cells (Conlon et al., 2016; Shan et al., 2017). The majority of research on persisters has been concentrated on the model organism *E. coli*, but it is widely recognized that many other medically relevant bacterial species may produce PCs (Moker et al., 2010; Keren et al., 2011; Lechner et al., 2012; Slattery et al., 2013). Aims of the present study were: (i) evaluate the ability of CCCP to induce persistence in cultures of *Staphylococcus aureus* and *Pseudomonas aeruginosa*, two bacterial species commonly implicated in relapsing and chronic infections; (ii) characterize the metabolic activity and the susceptibility to conventional antibiotics of CCCP-treated bacteria; (iii) investigate the susceptibility of CCCP-induced persisters to different membrane-targeting agents, including colistin, daptomycin and three structurally diverse AMPs. These included an optimized α-helical analog of temporin 1Tb (TB_L1FK) (Grassi et al., 2017), a β-sheet chimeric derivative of β-defensins (C5) (Jung et al., 2011) and a semi-synthetic dendrimeric peptide (Den-SB056) (Batoni et al., 2016). Indeed, since their main mechanism of action involves the permeabilization of bacterial membranes, AMPs are likely to exert a bactericidal activity also against metabolically dormant cells, thus emerging as promising candidates for the development of novel anti-persister drugs (Briers et al., 2014). Exposure to optimized concentrations of CCCP significantly increased the tolerance of *S. aureus* and *P. aeruginosa* to different classes of antibiotics and determined a global reduction in bacterial metabolic activity. The high antibiotic tolerance and reduction in metabolism, along with the reversion to a normal-growing and antibiotic-sensitive phenotype after CCCP removal, confirmed the development of the persister status in CCCP-treated cultures. Interestingly, CCCP-induced persisters of both bacterial species were susceptible to all tested AMPs at levels comparable to those of CCCP-untreated bacteria. Colistin was also quite active against persisters of *P. aeruginosa*, while daptomycin killed persisters of *S. aureus* only at high concentrations. Overall, CCCP treatment resulted to be a suitable method to induce persistence at high efficiency in medically relevant Gram-positive and Gram-negative bacteria and a useful tool to evaluate the anti-persister properties of novel antimicrobials. Unlike most conventional antibiotics, AMPs were able to kill PCs highlighting their potential employment as anti-persisters drugs.

## Materials and Methods

### Bacterial Strains and Culture Conditions

The reference laboratory strains *Pseudomonas aeruginosa* ATCC 27853 and *Staphylococcus aureus* ATCC 33591 were used in the study. Bacteria were grown in Luria-Bertani (LB) medium (Sigma-Aldrich, St. Louis, MO, United States) at 37°C with shaking for liquid cultures and on TSA (Oxoid, Basingstoke, United Kingdom) plates at 37°C for enumeration of colony forming units (CFU).

### Carbonyl Cyanide *m*-Chlorophenylhydrazone

Carbonyl cyanide *m*-chlorophenylhydrazone was purchased from Sigma-Aldrich. CCCP was diluted in DMSO to obtain a stock solution of 40 mg/mL and stored in aliquots at -20°C.

### Antibiotics

Ciprofloxacin, colistin, levofloxacin and meropenem were provided as purified powder by the manufacturer (Sigma–Aldrich). Daptomycin (Cubicin) was purchased as purified powder from Novartis (Basel, Switzerland). Gentamicin was supplied as a 10-mg/mL sterile solution by Sigma–Aldrich. A stock solution of 4 mg/mL of each antibiotic was prepared in sterile milli-Q water and stored in aliquots at -20°C.

### Antimicrobial Peptides

C5 and TB_L1FK were synthesized by Proteogenix (Schiltigheim, France). Den-SB056 was provided by PolyPeptide (Limhamn, Sweden). Analysis of synthetic peptides by high performance chromatography (HPLC) and mass spectrometry revealed purity ≥98%. Peptides were diluted in milli-Q water to obtain a stock solution of 1 mM and stored at -80°C. The main physicochemical features of the peptides are shown in **Table 1**.

**Table 1:** Main structural and physicochemical features of AMPs used in the study.

Peptide	Sequence	Molecular weight	Charge
C5	GIINTLQKYYCRVRGAICHPVFC	5382.84	+15
	PRRYKQIGKCSTRGRKCCRRKK
Den-SB056	[WKKIRVRLSA]_2_-K-8Aoc-NH_2_^a^	2749.76	+10
TB_L1FK	FLPIVGLLKSLLK-NH_2_	1440.86	+3

^a^*8-Aoc is 8-aminooctanoic acid, which is amidated in the peptide construct*.

### Evaluation of the Effect of CCCP on Cell Viability

The susceptibility of stationary-phase cultures of *P. aeruginosa* and *S. aureus* to CCCP was determined. Bacteria were grown in LB medium for approximately 18 h at 37°C to obtain stationary-phase cells, as assessed by monitoring bacterial growth by optical density. A volume of 1 mL of the overnight cultures was incubated with 10 μL of CCCP at different concentrations (from 25 to 400 μg/mL) for 3 h at 37°C with shaking. Bacterial cultures exposed to DMSO were used as cell viability control. Following the incubation, bacteria were washed twice in sodium-phosphate buffer (10 mM SPB, pH 7.4) by centrifugation (1700 × *g* for 10 min), subsequently diluted 10-fold in LB and plated on TSA to determine the number of CFU.

### Induction of Persistence with CCCP

Concentration of CCCP and exposure time able to efficiently induce persistence in stationary-phase cultures of *P. aeruginosa* and *S. aureus* were investigated. Overnight cultures of *P. aeruginosa* and *S. aureus* were incubated with different concentrations of CCCP (from 25 to 400 μg/mL) for 1 or 3 h at 37°C with shaking. The number of CCCP-induced persisters was determined based on their survival to antibiotic treatments (Kwan et al., 2013). To this end, after the pretreatment with CCCP, bacteria were washed twice in SPB (1700 × *g* for 10 min) and re-suspended in SPB/LB (SPB supplemented with 1% LB) at a final density of 1 × 10^6^ CFU/mL. Hence, bacterial suspensions were exposed to different antibiotics for 3 h: ciprofloxacin (5 μg/mL) or meropenem (10 μg/mL) in the case of *P. aeruginosa* and levofloxacin (2.5 μg/mL) or gentamicin (10 μg/mL) in the case of *S. aureus*. CCCP-untreated and CCCP-pretreated bacteria re-suspended in SPB/LB alone were used as cell viability controls. The antibiotic concentrations used in the experiments were chosen based on preliminary assays in which stationary-phase cultures of *P. aeruginosa* and *S. aureus* were treated with increasing concentrations of each antibiotic. The threshold concentrations at which a killing plateau was reached and only spontaneous PCs survived were used for the experiments (Fauvart et al., 2011). Exposure of CCCP-pretreated cells to antibiotics was performed in SPB/LB in order to avoid their metabolic reactivation and replication after CCCP removal. Following the antibiotic treatment, bacteria were plated on TSA and the number of surviving PCs was determined by CFU counting after 48 h of incubation at 37°C.

### Isothermal Microcalorimetry Assay

The metabolic profile of CCCP-induced persisters of *P. aeruginosa* and *S. aureus* was evaluated by real-time monitoring of bacterial heat production through isothermal microcalorimetry. A 48-channel isothermal microcalorimeter (Thermal Activity Monitor, Model 3102 TAM III, TA Instruments, New Castle, DE, United States) was used for the study. Microcalorimetry glass ampoules were aseptically filled with 1 mL of the stationary-phase cultures and 10 μL of CCCP at a final concentration of 200 and 400 μg/mL for *P. aeruginosa* and *S. aureus*, respectively. Ampoules were inserted into the calorimeter, placed in the thermal equilibration position for 15 min and then lowered into the measuring position. After 45 min of signal stabilization, measurements of the heat flow were initiated and recorded for 24 h. Data analysis was performed with the manufacturer software (TAM Assistant) and GraphPad Prism (GraphPad Software, La Jolla, CA, United States), and results were plotted as heat flow (in microwatts) or total heat (in joules) versus time.

### Flow Cytometry Assay

The metabolic activity of CCCP-induced PCs of *P. aeruginosa* and *S. aureus* was also evaluated by determining the oxidoreductase activity through flow cytometry. Stationary-phase cultures were incubated with CCCP at 200 and 400 μg/mL for *P. aeruginosa* and *S. aureus*, respectively. After a 3-h exposure, bacteria were washed twice in SPB (1700 × *g* for 10 min) and diluted to reach a cell density of 1 × 10^6^ CFU/mL. Then, bacterial suspensions were stained with the RedoxSensor^TM^ Green reagent (RSG) (BacLight^TM^ RedoxSensor^TM^ Green Vitality Kit, Thermo Fisher Scientific, Waltham, MA, United States) according to the manufacturer’s instructions. Briefly, 1 mL of the bacterial suspension was incubated with 1 μL (*P. aeruginosa*) or 0.1 μL (*S. aureus*) of 1 mM RSG for 10 min at 37°C in the dark. Stained samples were analyzed with a BD Accuri C6 flow cytometer (BD Biosciences, Mountain View, CA, United States) equipped with a 488 nm laser and a 533/30 nm optical filter. Data were collected and analyzed with BD Accuri C6 software (BD Biosciences), and results were plotted as cell counts versus fluorescence intensity.

### Revival Assay of CCCP-Induced Persisters

The time required for the revival of CCCP-induced persisters was determined by evaluating bacterial heat production and bacterial growth after the removal of CCCP. Furthermore, the susceptibility of CCCP-treated cells to different antibiotics after the revival was assessed in terms of MIC and MBC values according to the standard microdilution method and compared to that of untreated cultures. Briefly, stationary-phase cultures were incubated for 3 h with CCCP at 200 and 400 μg/mL for *P. aeruginosa* and *S. aureus*, respectively. Following incubation, bacteria were washed twice in SPB (1700 × *g* for 10 min) in order to remove CCCP, re-suspended in fresh LB and diluted to a final density of 1 × 10^6^ CFU/mL. Microcalorimetry ampoules were aseptically filled with 1 mL of the bacterial cultures and heat production was continuously determined for 24 h as previously described. In parallel, bacterial growth was monitored by measuring the optical density at 600 nm (OD_600_) of the bacterial cultures at different incubation times. Once the early exponential phase of growth was reached (OD_600_ of 0.5), bacteria were harvested, diluted in LB to a final density of 1 × 10^6^ CFU/mL and incubated into 96-well microplates with different concentrations of ciprofloxacin (0.125–2 μg/mL) or meropenem (2.5–40 μg/mL) in the case of *P. aeruginosa* and levofloxacin (0.125–2 μg/mL) or gentamicin (5–80 μg/mL) in the case of *S. aureus*. Bacterial cells suspended in LB alone were used as viability control. MIC values were defined as the lowest concentration of each antibiotic resulting in the complete inhibition of visible growth after 24 h of incubation at 37°C. For the determination of MBC values, bacteria from wells that did not exhibit growth were serially diluted and plated on TSA for CFU counting. The minimal concentration of antibiotic causing a CFU reduction of at least 3 Log_10_ compared to the initial inoculum was taken as the MBC European Committee for Antimicrobial Susceptibility Testing (EUCAST) of the European Society of Clinical Microbiology and Infectious Diseases (ESCMID) (2000). A number of 10 CFU/mL was taken as detection limit.

### Evaluation of the Activity of AMPs, Colistin and Daptomycin against CCCP-Induced Persisters

The activity of three different AMPs, colistin and daptomycin was tested against CCCP-induced persisters of *P. aeruginosa* and *S. aureus*. Persister-enriched cultures of *P. aeruginosa* and *S. aureus* were obtained by treatment with CCCP as described above. CCCP-treated and untreated bacteria were diluted in SPB/LB to a density of 1 × 10^6^ CFU/mL and incubated in the absence (viability control) or in the presence of different concentrations of C5, Den-SB056 and TB_L1FK (0.435–14 μM). Colistin (0.435–14 μM) and daptomycin (3.5–224 μM) were assayed against CCCP-treated cells of *P. aeruginosa* and *S. aureus*, respectively. For daptomycin testing, bacteria were diluted in deionized water supplemented with 1% LB and 75 μg/mL CaCl_2_. After 3 h of incubation at 37°C, samples were serially diluted and plated on TSA to determine the number of CFU.

### Statistical Analysis

All experiments were performed at least in triplicate and differences between mean values of groups were evaluated by one-way analysis of variance (ANOVA) followed by Tukey-Kramer post-hoc test. A *p*-value < 0.05 was considered statistically significant. Data analysis was performed with GraphPad InStat (GraphPad Software, La Jolla, CA, United States).

## Results

### Evaluation of the Effect of CCCP on Cell Viability

**FIGURE 1:**
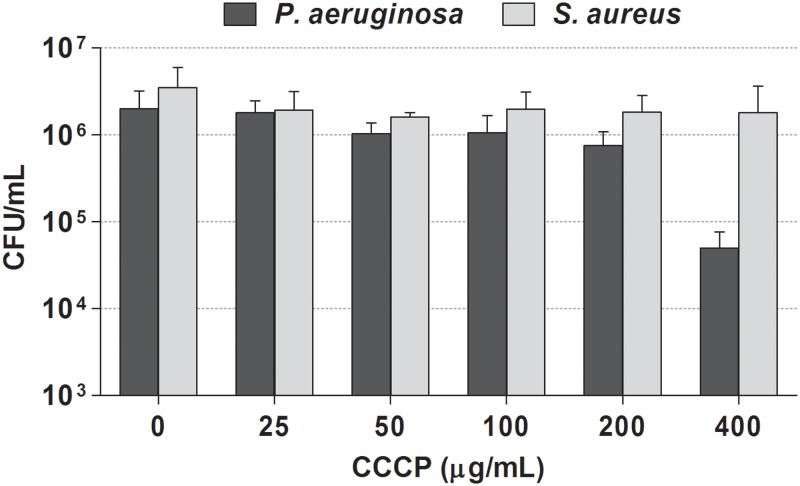
Effect of CCCP on viability of *P. aeruginosa* ATCC 27853 (black bars) and *S. aureus* ATCC 33591 (gray bars). Stationary-phase cultures of *P. aeruginosa* and *S. aureus* were exposed to different concentrations of CCCP for 3 h. Following the treatment, the number of surviving cells was evaluated by CFU counting. Data are reported as mean ± standard error of the mean of at least three independent experiments.

We first assessed the susceptibility of stationary-phase cultures of *P. aeruginosa* and *S. aureus* to CCCP in order to identify the concentrations able to ensure maximum cell survival. As shown in **Figure 1**, exposure of *P. aeruginosa* cultures to CCCP at concentrations up to 200 μg/mL did not exert a significant killing effect. In the case of *S. aureus*, only a minor effect on cell viability was observed by treating cultures with CCCP at concentrations up to 400 μg/mL.

### Generation of PCs of *P. aeruginosa* and *S. aureus* by CCCP Treatment

**FIGURE 2:**
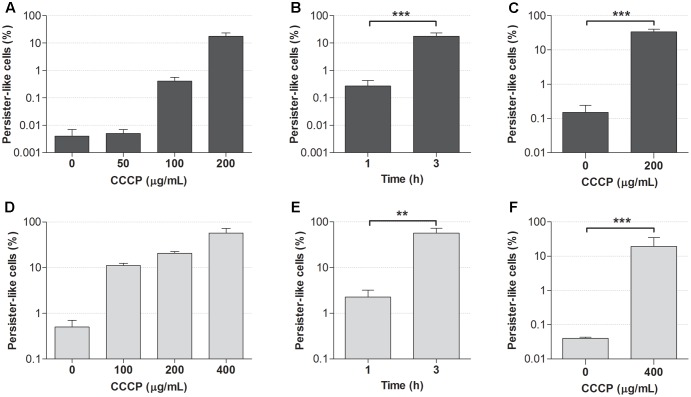
Effect of CCCP on the induction of persistence in stationary-phase cultures of *P. aeruginosa* ATCC 27853 **(A–C)** and *S. aureus* ATCC 33591 **(D–F)**. Ability of different concentrations of CCCP to induce a persister-like status was assessed by evaluating the percentage of cells surviving the treatment with ciprofloxacin (5 μg/mL) **(A)** or levofloxacin (2.5 μg/mL) **(D)** following 3-h exposure to CCCP. Optimal exposure time to CCCP was assessed based on cell survival to ciprofloxacin (5 μg/mL) **(B)** or levofloxacin (2.5 μg/mL) **(E)** following a pretreatment of 1 or 3 h with CCCP at 200 μg/mL (for *P. aeruginosa*) or 400 μg/mL (for *S. aureus*). Susceptibility of *P. aeruginosa* to meropenem (10 μg/mL) **(C)** and *S. aureus* to gentamicin (10 μg/mL) **(F)** was determined following a pretreatment of 3 h with CCCP. Data are reported as mean ± standard error of the mean of at least three independent experiments. ^∗∗^*p* < 0.01; ^∗∗∗^*p* < 0.001 (one-way ANOVA followed by Tukey-Kramer *post hoc* test).

The optimal concentration of CCCP and exposure time required for the induction of persistence in stationary-phase cultures of *P. aeruginosa* and *S. aureus* were established based on the acquisition of tolerance to antibiotics following the treatment with CCCP. Indeed, the lack of specific markers for persistence makes antibiotic tolerance the only reliable evidence of the persister status (Brauner et al., 2016). Two fluoroquinolones, ciprofloxacin (5 μg/mL) and levofloxacin (2.5 μg/mL), were initially used to assess the acquisition of antibiotic tolerance in CCCP-pretreated cells of *P. aeruginosa* and *S. aureus*, respectively. When these antibiotics were used against bacteria in the absence of CCCP pretreatment, only a very low percentage of the initial population survived antibiotic exposure (**Figures 2A,D**). In particular, less than 0.01% of stationary-phase cultures of *P. aeruginosa* survived ciprofloxacin exposure (**Figure 2A**), while approximately 0.5% of the initial inoculum of *S. aureus* tolerated levofloxacin treatment (**Figure 2D**), in accordance with the presence of naturally occurring PCs within the cultures. Interestingly, the percentage of bacteria surviving antibiotic treatment markedly increased in a dose-dependent manner following CCCP pretreatment. In the case of *P. aeruginosa*, cultures pretreated with CCCP at 200 μg/mL for 3 h showed a 5000-fold increase in the number of persister-like cells as compared to the untreated control (**Figure 2A**). In the case of *S. aureus*, the highest percentage of persister-like cells was obtained by pretreating cultures with CCCP at 400 μg/mL. Indeed, after a 3-h pretreatment, approximately 60% of the initial population survived levofloxacin exposure (**Figure 2D**). Furthermore, a time-dependent increase in the number of persister-like cells emerged from the evaluation of the antibiotic susceptibility of both bacterial species following a pre-incubation of 1 or 3 h with the identified optimal concentrations of CCCP. Indeed, a 3-h exposure to CCCP produced a statistically significant increase in the survival rate of *P. aeruginosa* and *S. aureus* to fluoroquinolones as compared to a pretreatment of 1 h (**Figures 2B,E**).

The ability of CCCP to induce persistence was confirmed by exposing CCCP-pretreated cultures to antibiotics with a different mechanism of action than fluoroquinolones, namely the β-lactam meropenem and the aminoglycoside gentamicin against *P. aeruginosa* and *S. aureus*, respectively. As shown in **Figures 2C,F**, stationary-phase cultures pretreated with CCCP for 3 h exhibited a significantly increased tolerance to meropenem and gentamicin as compared to the untreated controls. In particular, treatment of *P. aeruginosa* cultures with CCCP at 200 μg/mL determined a 200-fold increase in the percentage of bacteria tolerant to meropenem (35% of the starting bacterial population) (**Figure 2C**). Analogously, survival of *S. aureus* cultures to gentamicin increased by approximately 500 times upon exposure to CCCP at 400 μg/mL, resulting in 20% of the cells becoming persisters (**Figure 2F**).

Since a 3-h exposure to CCCP at 200 and 400 μg/mL resulted to be efficient in the induction of a persister-like status in *P. aeruginosa* and *S. aureus* respectively, these optimal conditions were adopted for the generation of persister-enriched cultures in all the subsequent experiments.

### Analysis of the Metabolic Activity of CCCP-Induced Persisters

**FIGURE 3:**
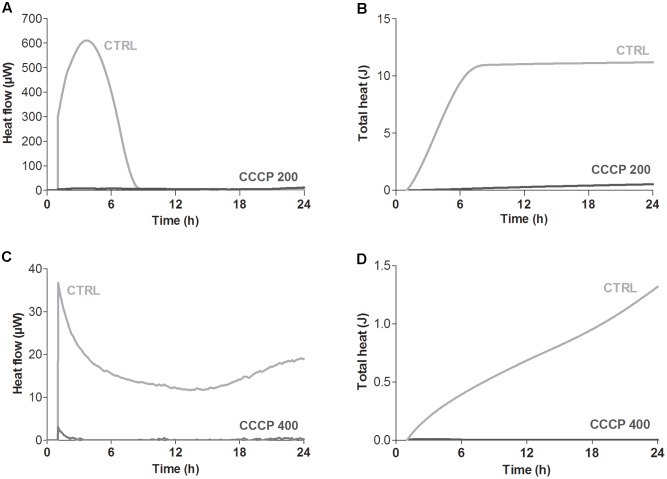
Global metabolic activity of CCCP-induced persisters of *P. aeruginosa* ATCC 27853 **(A,B)** and *S. aureus* ATCC 33591 **(C,D)**. Metabolic activity was evaluated by measuring in real time bacterial heat production through isothermal microcalorimetry for 24 h. Controls (CTRLs) represent CCCP-untreated stationary-phase cultures. Representative data of two independent experiments are reported.

Global metabolic activity of CCCP-treated cells of *P. aeruginosa* and *S. aureus* was quantitatively assessed in real time by isothermal microcalorimetry, a highly sensitive technique that allows to monitor microbial metabolic activity in the presence or absence of growth-inhibiting compounds through the measurement of heat production over time (Braissant et al., 2010; Di Luca et al., 2017). As shown in **Figure 3**, untreated stationary-phase cultures of both *P. aeruginosa* and *S. aureus* released heat over the time resulting in typical heat flow patterns. On the contrary, only a basal heat production was observed (<5 μWh) when both bacterial species were incubated with CCCP, suggesting an overall decrease in the metabolic activity of CCCP-treated cells.

**FIGURE 4:**
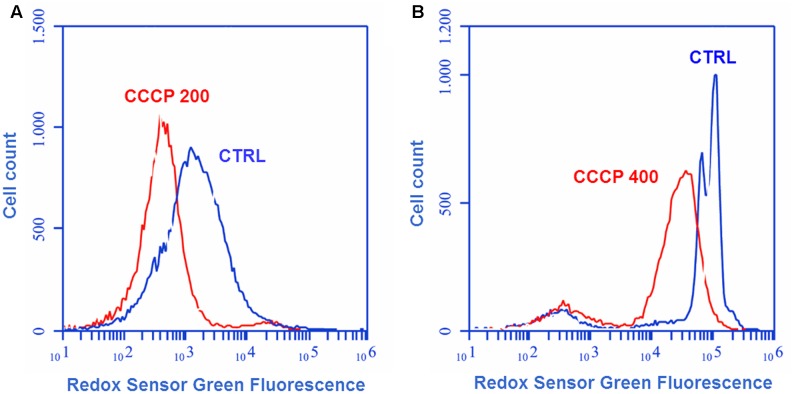
Oxidoreductase activity of CCCP-induced persisters of *P. aeruginosa* ATCC 27853 **(A)** and *S. aureus* ATCC 33591 **(B)**. Oxidoreductase activity of CCCP-pretreated cells was assessed through cytofluorimetric analysis after staining of the bacteria with the fluorogenic dye Redox Sensor Green (RSG). Controls (CTRLs) represent CCCP-untreated stationary-phase cultures. Representative data of two independent experiments are reported.

The effect of CCCP treatment on bacterial oxidoreductase activity was assessed through cytofluorimetric analysis after staining of the cells with the fluorogenic dye RSG. RSG has been recently reported to be a convenient indicator of metabolic activity at the single cell level since it provides a stable green fluorescent signal when reduced by bacterial reductases in metabolically active cells (Orman and Brynildsen, 2013; Orman et al., 2016). In the case of both *P. aeruginosa* (**Figure 4A**) and *S. aureus* (**Figure 4B**), a 3-h exposure to CCCP determined a reduction of approximately three times in RSG fluorescence as compared to the untreated control, indicating a drop in bacterial oxidoreductase activity following CCCP treatment.

### Evaluation of the Revival of CCCP-Induced Persisters

**FIGURE 5:**
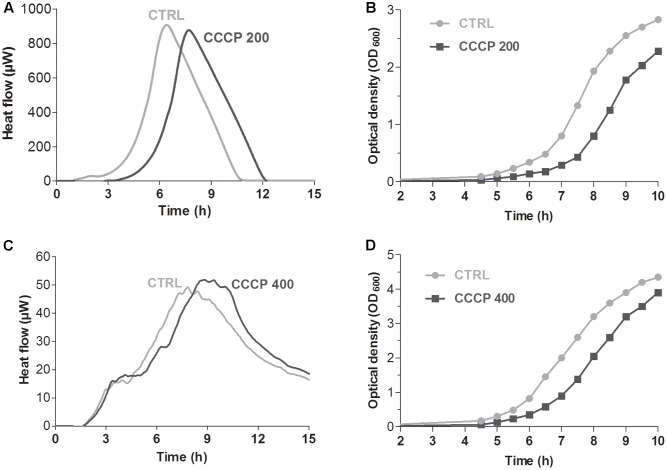
Revival of CCCP-induced persisters of *P. aeruginosa* ATCC 27853 **(A,B)** and *S. aureus* ATCC 33591 **(C,D)**. Time required for the revival of CCCP-induced persisters was determined by measuring bacterial heat production through isothermal microcalorimetry **(A,C)** and by monitoring bacterial growth by optical density **(B,D)** after the removal of CCCP and re-suspension of bacterial cells in fresh LB. CCCP-untreated bacteria incubated in fresh culture medium were used as controls (CTRLs). Representative data of two independent experiments are reported.

When incubated in a nutrient-rich environment, PCs have been shown to exhibit a characteristic lag phase before resumption of normal growth (Balaban et al., 2004; Gefen et al., 2008). Hence, in order to confirm further that CCCP pretreatment induced persistence, we evaluated the time required for the revival of the cultures upon removal of CCCP and exposure to fresh nutrients. The presence and duration of a time lag were initially determined by monitoring bacterial heat production through isothermal microcalorimetry. In the case of both *P. aeruginosa* (**Figure 5A**) and *S. aureus* (**Figure 5C**), identical heat flow patterns were obtained for CCCP-pretreated and untreated cultures. Nevertheless, CCCP-pretreated cells were characterized by a delay in heat production as compared to the untreated controls. Time of maximum heat production was extrapolated from the heat flow plots in order to determine the exact delay in the recovery of CCCP-pretreated bacteria. In this respect, a time lag of 1.5 h was recorded for CCCP-pretreated cultures of *P. aeruginosa* (**Figure 5A**), while a 1-h delay was observed in the case of *S. aureus* (**Figure 5C**). Similar results were also obtained when the revival was evaluated by measuring bacterial growth by optical density. Indeed, CCCP-induced persisters of both bacterial species displayed a lag phase of approximately 1 h before resuming replication at a normal rate (**Figures 5B,D**).

Susceptibility of CCCP-induced persisters to different classes of antibiotics was determined after the revival in order to verify the reversion of bacteria to a normal, antibiotic-sensitive phenotype. To this aim, the progeny of both CCCP-pretreated and untreated cells was harvested at early exponential growth phase and exposed to ciprofloxacin, gentamicin, levofloxacin or meropenem for the evaluation of the MIC and MBC values. As reported in **Table 2**, CCCP-pretreated and untreated cultures of both *P. aeruginosa* and *S. aureus* exhibited the same MIC and MBC values for each of the tested antibiotics, indicating that the loss of CCCP effect correlated with the sensitization to the action of antibiotics at the prior treatment levels.

**Table 2:** MIC and MBC values of ciprofloxacin, gentamicin, levofloxacin and meropenem against CCCP-pretreated and CCCP-untreated bacteria after their revival in fresh culture medium.

		CCCP-untreated		CCCP-pretreated	
		MIC	MBC	MIC	MBC
*P. aeruginosa*	Ciprofloxacin	0.5^a^	1	0.5	1
	Meropenem	10	10	10	10
*S. aureus*	Levofloxacin	0.5	0.5	0.5	0.5
	Gentamicin	20	40	20	40

^a^*Concentrations are expressed in μg/mL*.

### Evaluation of the Anti-persister Activity of AMPs and Conventional Peptide-Based Antibiotics

**FIGURE 6:**
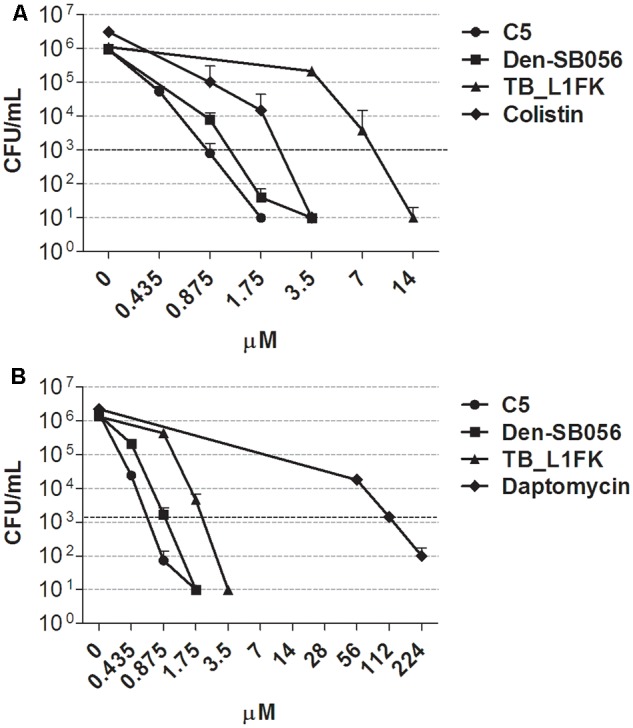
Activity of AMPs and peptide-based antibiotics against CCCP-induced persisters of *P. aeruginosa* ATCC 27853 **(A)** and *S. aureus* ATCC 33591 **(B)**. Anti-persister activity of C5, Den-SB056, TB_L1FK, colistin or daptomycin was evaluated by CFU counting after 1.5 h incubation with CCCP-pretreated cultures. Dashed lines indicate a reduction of 3 Log_10_ in the number of CFU of the starting inoculum. A number of 10 CFU/mL was taken as detection limit. Data are reported as mean ± standard error of the mean of at least three independent experiments.

The antimicrobial activity of three structurally different AMPs (**Table 1**) was determined by CFU counting against persister-enriched cultures of *P. aeruginosa* and *S. aureus* generated by CCCP treatment. Under the same experimental conditions, the anti-persister activity of the polypeptide colistin and the lipopeptide daptomycin was also assessed against CCCP-induced persisters of *P. aeruginosa* and *S. aureus*, respectively. In the case of both *P. aeruginosa* (**Figure 6A**) and *S. aureus* (**Figure 6B**), all three AMPs eradicated persister-enriched cultures to the limit of detection (10 CFU/mL) at concentrations ranging from 1.75 to 14 μM. Colistin displayed a bactericidal activity against CCCP-pretreated cells of *P. aeruginosa* at levels comparable to AMPs and determined the complete killing of the starting bacterial inoculum at concentrations as low as 3.5 μM (**Figure 6A**). On the contrary, a markedly reduced anti-persister activity against *S. aureus* was observed in the case of daptomycin, which exerted its bactericidal effect only at concentrations 32 to 64-fold higher than those of the tested AMPs (**Figure 6B**).

The susceptibility of CCCP-induced persisters to AMPs, colistin and daptomycin was compared to that of CCCP-untreated bacteria in terms of MBC values. As shown in **Table 3**, all tested AMPs and colistin exerted a bactericidal effect against CCCP-pretreated bacteria at levels comparable to those of CCCP-untreated bacteria. Indeed, the majority of the tested AMPs exhibited the same MBCs toward CCCP-pretreated and untreated cells of both *P. aeruginosa* and *S. aureus*. In the case of colistin, only a 2-fold increase in the MBC value against CCCP-pretreated bacteria of *P. aeruginosa* was recorded. Conversely, in the case of daptomycin, a 16-fold higher concentration was required to kill CCCP-induced persisters of *S. aureus* than that effective against untreated bacteria.

**Table 3:** MBC values of AMPs, colistin and daptomycin against CCCP-pretreated and CCCP-untreated cultures of *P. aeruginosa* ATCC 27853 and *S. aureus* ATCC 33591.

	*P. aeruginosa*		*S. aureus*	
	CCCP-untreated	CCCP-pretreated	CCCP-untreated	CCCP-pretreated
C5	0.87^a^	0.87	0.87	0.87
Den-SB056	0.87	1.75	1.75	1.75
TB_L1FK	14	14	7	3.5
Colistin	1.75	3.5	–	–
Daptomycin	–	–	7	112

^a^*Concentrations are expressed in μM*.

## Discussion

Over the last years, the importance of PCs in chronic and relapsing infections has been increasingly recognized and a growing body of research has focused on finding alternative strategies for the eradication of antibiotic tolerant bacteria. Several innovative approaches have been suggested for the elimination of PCs, including their reversion to a metabolically active and antibiotic-susceptible state through exposure to chemical compounds such as the fatty acid *cis*-2-decenoic acid and the quorum sensing inhibitor (*Z*)-4-bromo-5-(bromomethylene)-3-methylfuran-2(5*H*)-one (Kim et al., 2011; Pan et al., 2012; Marques et al., 2014). Considerable attention has been devoted to the identification of antimicrobial agents with a growth-independent mechanism of action in order to target both actively dividing cells and PCs. Indeed, eradication of entire bacterial populations is mandatory to avoid the relapse of the infection and minimize the chance of resistance development during prolonged antibiotic treatments (Zhang, 2014). In this regard, Conlon et al. (2013) have recently proposed a mechanism of persister killing based on activation of Clp protease and dysregulation of proteolysis by acyldepsipeptide-4 (ADEP4). Interestingly, combining ADEP4 with rifampicin resulted in complete eradication of *S. aureus* biofilms both *in vitro* and in a murine model of deep-seated infection (Conlon et al., 2013). Similarly, mitomycin C and cisplatin have been demonstrated to be active against a broad range of PCs due to the direct cross-linking of DNA (Kwan et al., 2015; Chowdhury et al., 2016). Anti-persister strategies involving cationic AMPs have also emerged as promising routes of investigation although still poorly explored. Due to their ability to physically damage bacterial membranes, AMPs have been proven effective in withstanding the dormant and quiescent state of PCs (Chen et al., 2011; Briers et al., 2014; Mohamed et al., 2016).

Although recent progress in developing alternative therapeutic strategies against PCs is encouraging, testing novel anti-persister molecules is often a complex process because of the difficulty in isolating the small fraction of PCs from the main bacterial population. Traditional isolation methods rely on the differential response of persister and non-persister subpopulations to antibiotic exposure, which results in typical biphasic killing patterns. Addition of increasing concentrations of antibiotics to bacterial cultures and/or prolonged exposure times determine a rapid elimination of non-persisters that is followed by a killing plateau ascribable to surviving PCs (Gefen and Balaban, 2009; Fauvart et al., 2011). Unfortunately, due to the low levels of naturally forming PCs, antibiotic-based methods provide persisters with a very low efficiency (Henry and Brynildsen, 2016). In addition, difficulties have been found in separating the surviving persister subpopulation from the large proportion of dead bacteria, which may interfere with the proper assessment of the antibacterial activity of molecules with unconventional mechanisms of action like membrane-active AMPs. Other more sophisticated protocols aimed at PCs analysis and isolation have been proposed (e.g., fluorescence-activated cell sorting based methods), but they are a feasible solution only for well-equipped laboratories (Orman and Brynildsen, 2013).

To address this issue, we sought to develop an improved and simple method for obtaining persister-enriched cultures of medically relevant bacteria based on the treatment with the uncoupling agent CCCP. The ability of CCCP to induce high levels of persistence has been previously reported for the model organism *E. coli* (Kwan et al., 2013). The development of a persister-like status in CCCP-treated cells has been linked to the inhibition of ATP synthesis and the consequent reduction in bacterial metabolic activity. Importantly, CCCP treatment mimics a naturally occurring mechanism of persister formation (i.e., ATP depletion), which has been recently demonstrated to control persistence in both *S. aureus* and *E. coli* (Conlon et al., 2016; Shan et al., 2017). Here we investigated the effect of CCCP on the induction of persistence in *P. aeruginosa* and *S. aureus* by evaluating the acquisition of antibiotic tolerance and the metabolic activity of CCCP-treated bacteria. Different parameters of the method were examined in order to define the optimal concentration of CCCP and exposure time able to ensure the maximum cell survival and to generate the highest number of PCs. We succeeded in obtaining high-persister cultures with only a minor effect on cell viability by treating stationary-phase cultures of *P. aeruginosa* and *S. aureus* for 3 h with CCCP at 200 and 400 μg/mL, respectively. Indeed, these exposure conditions determined a substantial increase in cell survival to the treatment with different classes of bactericidal antibiotics, inducing a persister status in 20–60% of the initial bacterial population. Tolerance to antibiotics was found to correlate with a significant decrease in bacterial metabolic activity as proven by isothermal microcalorimetry and flow cytometry. Although isothermal microcalorimetry is not yet routinely used in the field of microbiology, it emerged as a valuable technique for the assessment of the persister status in bacteria as it allowed to measure in real time bacterial metabolism in terms of heat production. Global reduction in bacterial heat production and low oxidoreductase activity confirmed the induction of a state of dormancy in CCCP-treated cells. Furthermore, since the exit of PCs from their dormant state is associated with a specific lag phase during the resuscitation process, we also evaluated the revival of CCCP-induced persisters upon incubation in a nutrient-rich medium (Gefen et al., 2008; Jõers et al., 2010). Like naturally occurring PCs, CCCP-induced persisters displayed a typical delay before resumption of normal growth, thus providing an additional indication of the efficiency of CCCP in inducing persistence. When tested for susceptibility to antibiotics, the bacterial population regrown from CCCP-induced persisters exhibited a normal and antibiotic-sensitive phenotype highlighting the transient nature of the induced persister status. Additionally, reversion to the normal phenotype allowed us to definitely characterize the antibiotic tolerance of CCCP-treated bacteria as persistence rather than spontaneous or CCCP-induced genetic resistance.

Persister-enriched cultures of *P. aeruginosa* and *S. aureus* generated by CCCP treatment were employed to evaluate the anti-persister activity of three structurally different AMPs. The polypeptide colistin and the lipopeptide daptomycin were also tested for their activity against PCs as a model of licensed peptide-based antibiotics with a membrane-targeting mechanism of action. All tested AMPs were highly effective against CCCP-induced persisters of both bacterial species, even leading to the complete eradication of the bacteria. Among the three peptides tested, C5 and Den-SB056 exhibited a superior bactericidal activity against both CCCP-treated and untreated cells as compared to TB_L1FK, reasonably due to their higher cationic charge and number of amino acid residues (Han et al., 2016). In the case of membrane-active antibiotics, we found colistin to be highly active against PCs of *P. aeruginosa*, in accordance with previous studies that have reported its ability to eradicate PCs of *E. coli* and to enhance the anti-persister activity of different antibiotics (Cui et al., 2016). Conversely, daptomycin was effective at eliminating CCCP-induced persisters of *S. aureus* only at very high concentrations (Lechner et al., 2012). Based on these observations, we were able to prove that our protocol ensures a reliable determination of the anti-persister activity of membrane-targeting molecules, allowing for the detection of the bactericidal activity of both highly active and poorly effective agents. Moreover, our results confirmed that targeting the bacterial membrane represents a valuable strategy to eliminate PCs. Antibiotic-tolerance of PCs can largely be explained by the arrest in metabolic activity since conventional antibiotics target active cellular processes like macromolecule biosynthesis. On the contrary, membrane-targeting molecules do not require an active metabolism to exert their bactericidal action, thus resulting in a rapid and effective killing of both replicating cells and non-replicating persisters (Briers et al., 2014; Cui et al., 2016). Therefore, membrane-active molecules may represent attractive templates for the development of novel anti-persister agents and a suitable treatment option for persistent biofilm-associated infections. Furthermore, the broad-spectrum anti-persister activity of AMPs constitutes an additional advantage of developing such molecules as anti-persister agents as they might be able to control multispecies infections involving both Gram-positive and Gram-negative bacteria.

## Conclusion

In the present study, we described an improved method for the generation of PCs based on CCCP treatment, which allowed to obtain bacterial cultures with high levels of persisters and only a negligible contamination from dead bacteria. Interestingly, our protocol resulted to be valid on different bacterial species emerging as a favorable tool for future studies on persistence and for testing the anti-persister activity of novel molecules against both Gram-positive and Gram-negative bacteria. Persister-enriched cultures obtained in the study made it possible to evaluate with sufficient accuracy the anti-persister properties of different membrane-targeting molecules revealing their potential as novel class of antimicrobials.

## Author Contributions

LG, MDL, GM, AR, SE, and GB: conception and design of the work; LG, MDL, GM, and SE: acquisition, analysis, and interpretation of the data; LG and GB: drafting of the work; LG, MDL, GM, AR, SE, AT, and GB: critical revision of the work; final approval.

## Conflict of Interest Statement

The authors declare that the research was conducted in the absence of any commercial or financial relationships that could be construed as a potential conflict of interest.

## Abbreviations

AMP: antimicrobial peptide
CCCP: carbonyl cyanide *m*-chlorophenylhydrazone
CFU: colony-forming units
DMSO: dimethyl sulfoxide
LB: Luria-Bertani Broth
MBC: minimal bactericidal concentration
MIC: minimal inhibitory concentration
PC: persister cell
RSG: redox sensor green
SPB: sodium-phosphate buffer
TSA: tryptone soy agar.
